# Microarray analysis of peripheral blood lymphocytes from ALS patients and the SAFE detection of the KEGG ALS pathway

**DOI:** 10.1186/1755-8794-4-74

**Published:** 2011-10-25

**Authors:** Jean-Luc C Mougeot, Zhen Li, Andrea E Price, Fred A Wright, Benjamin R Brooks

**Affiliations:** 1 Department of Neurology, ALS Biomarker Laboratory - James G Cannon Research Center, Carolinas Medical Center, 1542 Garden Terrace, Charlotte, NC 28203-6110, USA; 2 Department of Neurology, Carolinas Neuromuscular/ALS-MDA Center, Carolinas Medical Center, 1010 Edgehill Road North, Charlotte, NC 28207-1885, USA; 3 Department of Neurology, University of North Carolina School of Medicine-Charlotte Campus, Carolinas Medical Center, 1000 Blythe Blvd, Charlotte, NC 28203-5812, USA; 4 Department of Biostatistics, University of North Carolina at Chapel Hill, 4115B McGavran-Greenberg Hall, CB #7420, Chapel Hill, NC 27599-7420, USA

## Abstract

**Background:**

Sporadic amyotrophic lateral sclerosis (sALS) is a motor neuron disease with poorly understood etiology. Results of gene expression profiling studies of whole blood from ALS patients have not been validated and are difficult to relate to ALS pathogenesis because gene expression profiles depend on the relative abundance of the different cell types present in whole blood. We conducted microarray analyses using Agilent Human Whole Genome 4 × 44k Arrays on a more homogeneous cell population, namely purified peripheral blood lymphocytes (PBLs), from ALS patients and healthy controls to identify molecular signatures possibly relevant to ALS pathogenesis.

**Methods:**

Differentially expressed genes were determined by LIMMA (Linear Models for MicroArray) and SAM (Significance Analysis of Microarrays) analyses. The SAFE (Significance Analysis of Function and Expression) procedure was used to identify molecular pathway perturbations. Proteasome inhibition assays were conducted on cultured peripheral blood mononuclear cells (PBMCs) from ALS patients to confirm alteration of the Ubiquitin/Proteasome System (UPS).

**Results:**

For the first time, using SAFE in a global gene ontology analysis (gene set size 5-100), we show significant perturbation of the KEGG (Kyoto Encyclopedia of Genes and Genomes) ALS pathway of motor neuron degeneration in PBLs from ALS patients. This was the only KEGG disease pathway significantly upregulated among 25, and contributing genes, including *SOD1*, represented 54% of the encoded proteins or protein complexes of the KEGG ALS pathway. Further SAFE analysis, including gene set sizes >100, showed that only neurodegenerative diseases (4 out of 34 disease pathways) including ALS were significantly upregulated. Changes in *UBR2 *expression correlated inversely with time since onset of disease and directly with ALSFRS-R, implying that *UBR2 *was increased early in the course of ALS. Cultured PBMCs from ALS patients accumulated more ubiquitinated proteins than PBMCs from healthy controls in a serum-dependent manner confirming changes in this pathway.

**Conclusions:**

Our study indicates that PBLs from sALS patients are strong responders to systemic signals or local signals acquired by cell trafficking, representing changes in gene expression similar to those present in brain and spinal cord of sALS patients. PBLs may provide a useful means to study ALS pathogenesis.

## Background

Amyotrophic lateral sclerosis (ALS) is a progressive neurodegenerative disease causing muscle weakness and wasting resulting from the loss of motor neurons in brain and spinal cord characterized by ubiquitinated inclusions in brain and spinal cord of *post mortem *ALS patients [1]. Several genome-wide association studies (GWAS) have shown evidence of genetic heterogeneity underlying disease susceptibility [2]. Single nucleotide polymorphisms were found in the *ITPR2 *(inositol 1,4,5-triphosphate receptor, type 2) [3], *FGGY *(FGGY carbohydrate kinase domain containing) [4], *DPP6 *(dipeptidyl-peptidase 6) [5], with variable strength of association with ALS and limited replication. None of these genes has been proven relevant to the pathogenesis of ALS. More recently, mutations were found in the *UNC13A *(unc-13 homolog A) gene [6] and in the 9p21 chomosomal locus [7]. To overcome challenges in the interpretation of results from GWAS and data from the world of "omics" in general, ALS researchers are actively engaged in integrative global bioinfomatics and the creation of ALS models for development of new ALS therapies (Euro-MOTOR project) [8]. Despite genetic heterogeneity underlying disease susceptibility, the clinical manifestations of the ALS phenotype are relatively homogeneous; suggesting that at the cellular and molecular levels there may be a convergence of a limited number of pathways that could lead to the ALS phenotype.

Gene expression profiling studies using microarrays and/or real time quantitative RT-PCR have been conducted on various tissues from rodent models for ALS such as muscle or brain tissues, lumbar spinal anterior horn tissues, spinal cord motor neurons isolated by laser capture microdissection (LCM), whole blood or peripheral blood mononuclear cells (PBMCs). Similar studies were performed on spinal cord tissues or LCM-isolated motor neurons obtained *post mortem *from ALS patients. Roughly, ~1000 unique genes were found differentially expressed but only ~5% differentially expressed in the same direction in more than one study [9], indicating little reproducibility. Poor reproducibility may be due to the use of different gene expression profiling methods or platforms, tissue of different origin, methods used for biological sample preparation, time of tissue collection at pre-symptomatic or symptomatic stage, and use of a particular batch of rodents or human cohort. Rather, one may find greater commonalities at the pathway alteration level with regard to apoptosis regulation, calcium regulation, oxidative stress and mitochondrial function, ER-stress and unfolded protein response (UPR), UPS and autophagy, RNA processing, DNA metabolism, axonal transport, integrity of the neuromuscular junction, muscle atrophy, and direct/indirect interactions with astrocytes, microglia and T-cells. Within these biological processes, genes of importance are those with mutations or polymorphisms shown to confer susceptibility to or cause ALS; or genes playing a critical role in the pathways that involve susceptibility genes.

A number of studies have sought blood biomarkers that may be useful to detect early signs of ALS, assess disease progression, monitor treatment effects, or track down the cause(s) of the disease, in a minimally-invasive fashion in ALS patients. Using qRT-PCR, Lin *et al. *(2009) have shown subtle transcriptional down-regulation of mitochondrial electron-transfer chain genes in whole blood from ALS patients [10]. Saris *et al. *(2009) have identified co-expressed gene modules (clusters) in total blood from sporadic ALS (sALS) patients [11]. These findings resulted from subtle differential expression of 2300 probe-encoded genes and were related to biological/disease categories such as post-translational modification, infection mechanism, inflammatory disease, neurological disorder, and skeletal and muscular disorder. Gagliardi *et al. *(2010) showed increased *SOD1 *mRNA expression in spinal cord, brain stem and lymphocytes of sporadic ALS (sALS) patients [12]. Zhang *et al. *(2011) identified gene expression profiles of short-term cultured PBMCs from ALS patients, demonstrating the activation of monocytes/macrophages *via *the LPS/TLR4 neuroinflammatory pathway [13]. Lincecum *et al. *(2010) demonstrated the activation in ALS pathogenesis of a co-stimulatory pathway bridging the activation of T-cell responses and the amplification of the innate immune response, based on gene expression profiles obtained from whole blood of the G93A SOD1 mouse model and ALS patients [14]. Circulating white blood cells might acquire certain properties from long distance signals mediated by small metabolites or macromolecules circulating in peripheral blood. They might also acquire novel properties from trafficking at sites of neurodegeneration associated with rupture of the blood brain barrier or blood-spinal cord barrier in early and late ALS to a variable degree. Further investigation in this area of ALS research is critically needed [15].

In the current work, we analyzed RNA extracted from PBLs of ALS patients and control subjects, thereby reducing some of the complexity of mixed expression patterns generated by RNA from reticulocytes, granulocytes, monocytes, thrombocytes and plasma, normally present in whole blood. Indeed, gene expression profiles of blood-derived samples are strongly dependent on the predominant constituent cell type(s) [16,17]. Analyses of mRNA expression data by LIMMA [18], SAM [19] and SAFE [20], revealed alterations of the ubiquitin/proteasome system (UPS). Using proteasome inhibition assays, parallel changes of UPS activity at the protein level were determined in subcultured PBMCs (mainly composed of lymphocytes) from ALS patients, by Western blot analysis.

## Methods

### Isolation of peripheral blood lymphocytes from ALS patients and controls

During year 2007 until March 2008, blood samples to be used for microarray analysis were collected at Carolinas Neuromuscular/ALS-MDA Center with approval by the IRB at Carolinas Medical Center. Informed consent was obtained from all participants to this study. ALS diagnosis was determined according to the El Escorial Criteria for "definite" ALS after exclusion of other conditions [21]. Disease onset was defined as time of initial weakness, dysarthria or dysphagia. Blood samples (~18 mL) were drawn from sporadic definite ALS patients and healthy control (HC) subjects by venipuncture into tubes adequate for either serum or lymphocyte isolation. The healthy controls (HCs) consisted of 9 white females (mean age 51.4 ± 11 (standard deviation) years) and 2 white males (64, 65). The sALS patients consisted of one black male (49), one black female (69), 5 white females (mean age 59 ± 20 years), and 4 white males (mean age 47 ± 9 years). Table 1 presents the clinical characteristics of the enrolled patients and healthy controls subjected to microarray analysis. PBMCs were isolated using Histopaque™-1077 density gradient centrifugation method. Using this procedure, yields were generally 1-2 × 10^6 ^PBMCs per mL of blood. Lymphocytes were further enriched to over 90% purity from the PBMC fraction by subsequent PERCOLL gradient centrifugation [22]. Blood samples were processed immediately upon reception in the lab within 30 minutes after blood draw.

**Table 1:** Demographic and clinical data for ALS patients (n = 11) and healthy controls (n = 11) enrolled for Agilent Human Whole Genome 4 × 44k Array analysis

Clinical Data at Time of Collection	ALS patients	Healthy controls
Mean age ± SD	53.8 ± 13	52.2 ± 11
Female	6	9
Male	5	2
Bulbar onset	2	-
Limb onset	8	-
Generalized	1	-
ALSFRS-R <24	5	-
ALSFRS-R >24	6	-
Onset of weakness ≤1 yr	4	-
Onset of weakness 1-5 yrs	4	-
Onset of weakness >5 yrs	3	-
Mean age of onset ± SD	47.2 ± 18	-
Death <3 yrs post-onset	3	-
Death >5 yrs post-onset	2	-
Mean age of death (n = 5) ± SD	62.2 ± 12	-

Three subjects with an ALSFRS-R >24 died within three years (1.5, 2, and 2.5 years) following ALS onset, and two others with an ALSFRS-R <24 died beyond 5 years (7.5 and 9.5 years). Some ALS patients were treated with riluzole (5 out of 11) and taking dietary supplements (7 out of 11) compared to healthy controls which are not matched in this regard. SD is standard deviation.

### RNA extraction, amplification, and dual mode reference design microarrays

The common reference design [23] was used for sample assignment in the dual color mode of expression assay on the Agilent Human Whole Genome 4 × 44k Microarrays to analyze ~40000 transcripts. Microarray experiments were performed, in which each of the 22 RNA samples (HC and sALS) was co-hybridized with RNA from the HC reference pool that was constituted with equal amounts of each of the 11 RNA samples from healthy controls. Total RNA stored in TRIzol (Invitrogen) at -80°C, was extracted from the lymphocyte samples at Cogenics, Inc. (Morrisville, NC) by standard procedures. The quantity of each of the total RNA samples and determination of the A_260/280 nm _ratio was determined by spectrophotometry and the size distribution was assessed using an Agilent Bioanalyzer. Fifty nanograms of total RNA was converted into labelled cRNA with nucleotides coupled to a fluorescent dye (either Cy3 or Cy5) using the Quick Amp Kit (Agilent Technologies, Palo Alto, CA) following the manufacturer's protocol. The A_260/280 nm _ratio and yield of each of the cRNAs were determined and a quality assessment was done using an Agilent Bioanalyzer. Equal amounts of Cy3 and Cy5-labeled cRNA (825 ng) from two different samples were hybridized to Agilent Human Whole Genome 4 × 44k Microarrays. The hybridized array was washed and scanned and data were extracted from the scanned image using Feature Extraction version 10.2 (Agilent Technologies). The non-normalized and normalized microarray datasets have been deposited in the NCBI Gene Expression Omnibus [24] as series GSE28253.

### Statistical analyses and SAFE data mining

Raw data .txt files in Agilent format were converted to .MEV files using ExpressConverter™ v2.1 of the TM4 Microarray Suite (TIGR Genomics, Rockville, CA). Background-subtracted raw data were normalized using the MIDAS pipeline (TM4, TIGR Genomics, Rockville, MD) according to Sioson *et al. *(2006) with the following steps: total intensity normalization, LocFit (LOWESS), standard deviation regularization and low intensity trim [25]. Filtering stringencies requiring that the integrated signal intensities (ISI) for each Cy3 and Cy5 channels were more than two standard deviation(s) of the Cy3 and Cy5 background (ISI = 7000), generated the dataset DS7000 [7199 probes, 5540 unique genes]. DS7000 was subjected to LIMMA [18] and SAM [19] analyses using TMeV v4.5.1 program (TM4, TIGR Genomics, Rockville, MD) to determine differentially expressed genes. The false discovery rate (FDR) was 1.17% (delta = 0.90) for DS7000. SAFE analysis was performed with Bioconductor 2.5 according to Barry, Nobel, and Wright (2005) [20] to identify gene sets demonstrating different expression levels between classes of comparison. Default settings for local (*t-test*) and global (Wilcoxon) statistics were used. Comparisons were based on gene ontology databases for biological processes, molecular functions, cellular components, and the protein families (Pfam) and KEGG databases.

### Total ubiquitination and proteasome inhibition assays with PBMCs from ALS patients and healthy controls

Freshly isolated PBMCs (composed of ~80% lymphocytes and ~20% of monocytes per flow cytometry analysis) from ALS patients and healthy controls, were subcultured overnight (O.N.) for 18 hr at 37°C in RPMI (Invitrogen) supplemented with 10% FCS (Invitrogen) and supplemented or not with added-back matched autologous serum that had been prepared in parallel. These PBMCs were treated, or not treated, for 1.5 hrs at 37°C with the reversible proteasome inhibitor MG132 (Sigma). For each patient or healthy control, serum was prepared separately from PBMCs from the same blood draw. Matched serum was supplemented at a concentration of 20% to the O.N. cultures. A total of 750000 cells were seeded per well of a 48-well plate. Cells were treated with 10 μM proteasome inhibitor MG132 or DMSO vehicle at 0.09% for 1.5 hrs. Cells were collected and snap frozen until Western blot analysis. Cells were lysed into RIPA buffer (150 mM NaCl, 1.0% IGEPAL^® ^CA-630, 0.5% sodium deoxycholate, 0.1% SDS, 50 mM Tris, pH 8.0) including protease inhibitor cocktails [complete Mini EDTA-free protease inhibitor cocktail tablets (Roche) and Protease Inhibitor cocktail P8340 (Sigma)]. Total protein was quantified using BioRad Dc protein assay. An aliquot (20 μg total protein) was supplemented with 2x Laemmli's sample buffer and boiled for 5 minutes prior to separation by sodium dodecyl sulfate (SDS)-12.5% polyacrylamide gel electrophoresis for 130 min at 100V. Proteins were transferred to polyvinylidene difluoride membranes (Millipore) and quenched with blocking buffer containing 10% non-fat milk in PBS-0.1 Tween 20 for 1 hr at room temperature. The membranes were incubated overnight at 4°C with primary monoclonal anti-ubiquitin sc-8017 antibody (1/1000 dilution; Santa Cruz) diluted in the blocking buffer. Membranes were subsequently incubated with goat anti-mouse human absorbed HRP-secondary sc-2055 antibody (1/10000 dilution; Santa Cruz) for 30 minutes and assayed using the Super Signal Pico chemiluminescence detection system (Thermo Fisher Scientific). Subsequent reprobing with anti-beta-actin antibody sc-81178 (Santa Cruz) was performed by stripping membranes of bound antibodies in stripping buffer (62.5 mM Tris HCL, 2% SDS, and 100 mM 2-mercaptoethanol [pH 6.7]) at 56°C for 20 minutes. ECL films and a LAS3000 imaging system (Fuji) were used for detection of the chemiluminescence. Silver staining was used to confirm loading homogeneity in the PAGEs post-electrotransfer using SilverSNAP stain (Thermo Fisher Scientific), in addition to reprobing of the membranes for beta-actin.

### Semi-quantitative analysis of the Western blot data

Raw images were processed in ImageJ program (Dr. Wayne Rasband, wayne@codon.nih.gov, National Institute of Mental Health, Bethesda, Maryland, USA). The accumulated HMW ubiquitinated protein forms were delineated by a rectangular area, for which the background subtracted integrated density could be measured. The integrated density could then be measured for same area below the accumulated forms at a level of the blotting membrane demonstrating consistency of staining throughout the lanes, thereby providing a contrast reference area per lane. Calculation of a signal-to-noise (S/N) ratio for the accumulated forms was then determined independently from the detection of beta-actin that was achieved by stripping and reprobing the Western blot membranes.

## Results

**Figure 1:**
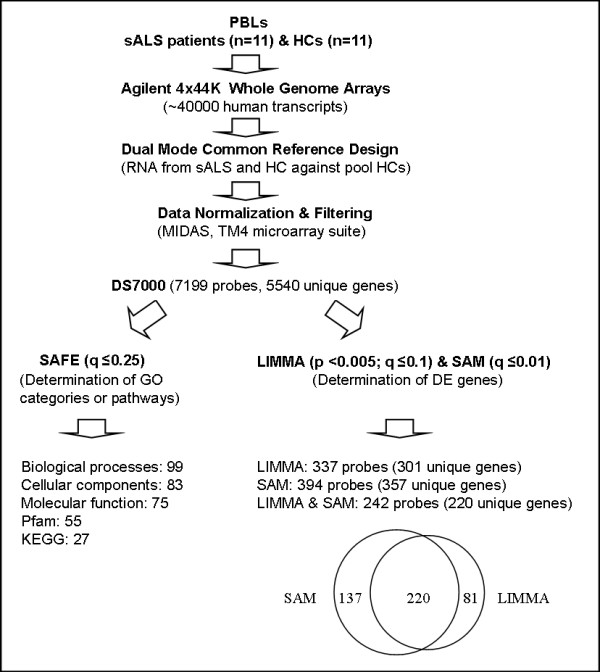
**Normalization, filtering, SAFE, LIMMA and SAM analyses of lymphocyte-derived microarray data from ALS patients and healthy controls**. Microarray analyses were performed on purified PBLs isolated from patients affected by sporadic amyotrophic lateral sclerosis (sALS) (n = 11) and healthy control subjects (HCs) (n = 11). The dual color mode in the common reference design was used to interrogate the expression of ~40000 transcripts (~30000 unique genes) using Agilent Human Whole Genome 4 × 44k Microarrays. Raw expression data were normalized and filtered using the MIDAS pipeline in the TM4 microarray suite (TIGR Genomics, Rockville, MD) to generate the dataset DS7000. SAFE was used for testing enrichment of functional gene ontology (GO) categories related to biological processes, molecular functions, cellular components, protein families (Pfam) and the KEGG databases. DS7000 was subjected to LIMMA and SAM analyses using TM4/TMeV v4.5.1 to determine differentially expressed (DE) genes. The online tool Data Overlapping and Area-Proportional Venn Diagram (http://bioinforx.com/free/bxarrays/overlap.php) was used to generate the Venn diagram.

We studied gene expression profiles of lymphocytes isolated from 11 patients diagnosed with definite sporadic ALS (sALS) and 11 healthy control subjects. Clinical characteristics for this cohort are described in Table 1. Figure 1 summarizes results from microarray data normalization and LIMMA, SAM and SAFE analyses.

### LIMMA and SAM analyses

Differentially expressed genes between ALS patients and healthy controls were determined using LIMMA (p_LIMMA _< 0.001, q_LIMMA _≤ 10%) and SAM (q_SAM _≤ 1%) in TM4/TMeV v4.5.1 program [25] for the dataset DS7000 (Figure 1). A significant overlap was found by comparing LIMMA and SAM results (Figure 1, Additional File 1). Table 2 presents the 24 most differentially expressed genes (q_SAM _= 0%, p_LIMMA _< 0.0005, q_LIMMA _≤ 10%). Five genes (*C12orf35*, *DYNLT1*, *IRS2*, *SKIV2L2*, and *TARDBP*) were significant at high stringency (q_SAM _= 0%, p_LIMMA _< 0.001, q_LIMMA _< 5%). *C12orf35*, *DYNLT1*, *SKIV2L2*, and *TARDBP *were upregulated 1.5-1.8 fold change (FC), while *IRS2 *was downregulated by two fold. *DYNLT1 *(dynein, light chain, Tctex-type 1) encodes a component of the dynein-dynactin complex composed of dynactin (*DCTN1*), those mutations have been involved in ALS [26], while *TARDBP *encodes TAR DNA binding protein 43 (TDP-43), those mutations may cause ALS [27]. *TIA-1*, a marker of stress granules that colocalizes with TDP-43 inclusions in frontotemporal lobar degeneration (FTLD-U) and ALS [28], is also upregulated (FC = 1.4, q_SAM _= 0%, p_LIMMA _< 0.005, q_LIMMA_~10%; Additional File 1. *SKIV2L2 *encodes a DEAD-box RNA helicase which is part of the exosome and spliceosome complexes [29,30] and it is known, for example, that some DEAD-box RNA helicases interact with *FUS/TLS *to control pre-mRNA splicing [31]. Defects or deregulation in RNA processing are a hallmark in the pathogenesis of motor neuron diseases, since motor neurons may be uniquely sensitive to perturbations in RNA processing pathways [32]. We also note that *IL7R *(interleukin-7 receptor subunit alpha), polymorphisms of which have been associated with risk in multiple sclerosis [33], is upregulated (FC = 1.7, q_SAM _= 0%, p < 0.005, Additional File 1). In addition, we identified upregulation (FC = 1.4, q_SAM_~0.5%, p_LIMMA _< 0.005, q_LIMMA_~10%) of *RTN4IP1 *(reticulon 4 interacting protein *alias *NOGO-interacting mitochondrial protein), a mitochondrial protein that interacts with reticulon 4, a potent inhibitor of regeneration following spinal cord injury [34]. We also identified upregulation of *SOD1 *(FC = 1.3, q~1%, p_LIMMA_<0.05, q_LIMMA_~10%), confirming the work by Gagliardi *et al. *(2010) [12].

**Table 2:** Differentially expressed genes in peripheral blood lymphocytes from ALS patients by SAM (q = 0) ranked by independent LIMMA (p < 0.0005)

^**α**^**Probe**	^**β**^**Symbol**	^**γ**^**Gene Description**	^**δ**^**p value**	^**ε**^**q value**	^**χ**^**FC**
A23P8185	*DYNLT1*	dynein, light chain, Tctex-type 1	0.000004	0.028	1.5
A24P154037	*IRS2*	insulin receptor substrate 2	0.000015	0.048	0.5
A32P234935	*TARDBP*	TAR DNA binding protein	0.000024	0.048	1.6
A23P98930	*C12orf35*	chromosome 12 open reading frame 35	0.000031	0.048	1.6
A23P110661	*SKIV2L2*	superkiller viralicidic activity 2-like 2	0.000033	0.048	1.8
A23P104624	*ENDOD1*	endonuclease domain containing 1	0.000069	0.083	1.5
A23P317800	*ANAPC4*	anaphase promoting complex subunit 4	0.000106	0.100	1.4
A23P21673	*KIAA1797*	KIAA1797	0.000150	0.100	1.5
A23P145437	*PHIP*	pleckstrin homology domain interacting protein	0.000185	0.100	1.4
A23P15714	*NSF*	N-ethylmaleimide-sensitive factor	0.000210	0.100	1.6
A23P82588	*C7orf55*	chromosome 7 open reading frame 55	0.000234	0.100	1.6
A23P120153	*RNF149*	ring finger protein 149	0.000290	0.100	1.7
A23P70998	*tcag7.903*	full-length cDNA clone CS0DF028YG12	0.000308	0.100	1.5
A23P145874	*SAMD9L*	sterile alpha motif domain containing 9-like	0.000310	0.100	2.1
A24P172481	*TRIM22*	tripartite motif-containing 22	0.000319	0.100	1.5
A23P42664	*SHFM1*	split hand/foot malformation (ectrodactyly) type 1	0.000328	0.100	1.6
A23P156355	*TMEM161B*	transmembrane protein 161B	0.000340	0.100	1.5
A23P129925	*SLFN11*	schlafen family member 11	0.000364	0.100	1.5
A24P209455	*GIMAP4*	GTPase, IMAP family member 4	0.000383	0.100	1.8
A23P84775	*PLRG1*	pleiotropic regulator 1	0.000386	0.100	1.5
A23P213255	*SMARCAD1*	SWI/SNF-related, matrix-associated actin-dependent regulator of chromatin, subfamily a, containing 1 DEAD/H box 1	0.000445	0.100	1.6
A23P19565	*ASCC3*	activating signal cointegrator 1 complex subunit 3	0.000457	0.100	1.4
A23P61854	*KIAA0372*	tetratricopeptide repeat protein 37	0.000461	0.100	1.4
A23P91891	*COPB2*	coatomer protein complex, subunit beta 2	0.000471	0.100	1.9

The list presents the 24 most discriminatory genes distinguishing the definite sALS patients (n = 11) from the healthy control subjects (n = 11). ^α^Agilent Array 4 × 44K probe ID, ^β^gene symbol, ^γ^description, ^δ^LIMMA significance p value, ^ε^LIMMA FDR (q value), and ^χ^fold change (FC) in expression are indicated. Using the TM4-MIDAS/TMeV pipeline, all genes had a local FDR of q = 0 according to SAM performed on DS7000 [7199 probes, 5540 unique genes]. Five genes had a LIMMA q value ≤0.05: *DYNLT1*, *IRS2*, *TARDBP*, *C12orf35*, and *SKIV2L2*.

### SAFE identification of molecular signatures in lymphocytes from ALS patients

SAFE [20] is a resampling-based procedure that is similar to GSEA (Gene Set Enrichment Analysis) [35], but with more flexible choices of test statistics. SAFE was used to obtain information on unifying biological themes from databases specific for (i) gene ontology (GO) pathways/categories (biological process, cellular component and molecular function), (ii) pathways/categories defined by the KEGG (Kyoto Encyclopedia of Genes and Genomes) and (iii) Pfam (protein families). Such resampling procedures have been shown to provide more accurate control of false positives than simpler enrichment-test methods using only lists of p-values [36].

Following determination of local (*t-test*) and global (Wilcoxon test) statistics using SAFE default settings, the significance for each gene set category was determined by bootstrap re-sampling and multiple test correction (for the multiple categories examined) by an FDR procedure with q_SAFE _< 25% considered significant (similarly to GSEA). This relatively liberal threshold was intended to avoid false negatives, although many of the findings presented here achieve more striking significance. For SAFE gene ontology category analysis, gene sets of 5-100 genes were examined, similar to restrictions used by others *(e.g.*, Barry *et al.*, 2005) [20]. This approach ensures that gene sets were not so small as to call into question a "pathway" interpretation, and not so large as to defy biological interpretation. In addition, the approach helps to manage the multiple testing penalties across numerous categories. To simplify overall interpretation, we only reported "upregulated" categories to highlight pathway activations caused by the disease rather than pathway inhibitions.

Significant upregulated categories representing gene sets associated with "biological processes", "cellular components", and "KEGG pathways" are shown in Tables 3, 4 and 5. We used same gene set size restriction (5-100 genes) for SAFE analysis of KEGG gene ontology groups including the KEGG Human Disease pathways, of which 25 were annotated in Bioconductor 2.5 for the Agilent platform. We identified that the KEGG ALS pathway was significant (q_SAFE _= 18%). The KEGG ALS pathway was the only significantly upregulated disease pathway among the 25 disease pathways (Additional File 2). In addition, we considered all 34 KEGG Human Disease pathways annotated in Bioconductor 2.5 for the Agilent platform among 55 total represented in the KEGG database (http://www.genome.jp/kegg-bin/get_htext?htext=br08901&query=%22Human%20Diseases%22&option=-s) [37] by including gene set sizes >100 in a secondary analysis.

**Table 3:** SAFE gene ontology pathways related to Biological Processes affected in peripheral blood lymphocytes from ALS patients

^**α**^**Pathway**	^**β**^**Sizes**	^**δ**^**q-value**	^**ε**^**GO Term**
**DNA Metabolism**			
GO:0006310	54/71	0	DNA recombination
GO:0006302	27/50	0.003	double-strand break repair
GO:0000718	12/21	0.0414	nucleotide-excision repair DNA damage removal
**ER and Golgi**			
GO:0006895	6/7	0.0071	Golgi to endosome transport
GO:0006888	26/43	0.1127	ER to Golgi vesicle-mediated transport
GO:0006904	10/19	0.0071	vesicle docking during exocytosis
GO:0006892	11/41	0.0471	post-Golgi vesicle-mediated transport
GO:0007041	7/6	0.1885	lysosomal transport
**Mitochondrial Function**			
GO:0022904	53/98	0.0558	mitochondrial ATP synthesis coupled electron transport
			respiratory electron transport chain
GO:0006120	44/43	0.0523	mitochondrial electron transport NADH to ubiquinone
GO:0006626	17/25	0.0972	protein targeting to mitochondrion
GO:0006119	81/8	0.1	oxidative phosphorylation
**Neurological Function**			
GO:0010001	8/13	0.1	glial cell differentiation
GO:0042552	8/30	0.1138	myelination
**Oxidation**			
GO:0019395	19/6	0.2252	fatty acid oxidation
**RNA Metabolism**			
GO:0000387	25/26	0.003	spliceosomal snRNP biogenesis
GO:0033119	10/3	0.0071	negative regulation of RNA splicing
GO:0051028	81/62	0.0644	mRNA transport
**PTM**			
GO:0070206	7/7	0	protein trimerization
GO:0018279	8/47	0.0071	protein amino acid N-linked glycosylation via asparagine
GO:0051262	10/12	0.0644	protein tetramerization
GO:0006465	7/9	0.0835	signal peptide processing
GO:0045116	8/7	0.0644	protein neddylation
GO:0016925	15/11	0.0627	protein sumoylation
**UPS**			
GO:0051443	62/6	0.0644	positive regulation of ubiquitin-protein ligase activity
GO:0051444	59/4	0.0644	negative regulation of ubiquitin-protein ligase activity
GO:0043161	79/43	0.0644	proteasomal ubiquitin-dependent protein catabolic process
**Viral Infection**			
GO:0019047	11/8	0.044	provirus integration
GO:0019059	18/12	0.0852	initiation of viral infection
GO:0019058	41/92	0.0644	viral infectious cycle

^α^Gene ontology (GO) pathway identities for biological processes and ^β^number of probes represented on the 4 × 44K human genome array per GO group of directly associated proteins is shown (note: one gene may be represented by different or redundant probes on the array). ^γ^FDR (q-value) at significance level p < 0.25 (25%) was determined by bootstrapping following SAFE analysis. ^δ^The table shows partial listing of representative significant ^ε^GO categories associated with biological processes (30 among 99). UPS is ubiquitin/proteasome system, PTM is post-translational modification, and ER is endoplasmic reticulum.

**Table 4:** SAFE gene ontology pathways related to Cellular Components affected in peripheral blood lymphocytes from ALS patients

^**α**^**Pathway**	^**β**^**Sizes**	^**δ**^**q-value**	^**ε**^**GO Term**
**Cytoskeleton**			
GO:0005868	6/10	0.001	cytoplasmic dynein complex
GO:0005885	8/7	0.0322	Arp2/3 protein complex
**ER and Golgi**			
GO:0030130	9/9	0.0001	clathrin coat of trans-Golgi network vesicle
GO:0008250	8/10	0.0001	oligosaccharyltransferase complex
GO:0030660	23/6	0.0001	Golgi-associated vesicle membrane
GO:0030134	7/3	0.0057	ER to Golgi transport vesicle
GO:0005791	12/20	0.0613	rough endoplasmic reticulum
GO:0030131	18/19	0.024	clathrin adaptor complex
GO:0030119	18/5	0.024	AP-type membrane coat adaptor complex
GO:0001669	8/34	0.2372	acrosomal vesicle
GO:0030127	6/8	0.0122	COPII vesicle coat
GO:0030126	8/12	0.003	COPI vesicle coat
**Mitochondria**			
GO:0005758	22/30	0.0004	mitochondrial intermembrane space
GO:0005763	20/18	0.0002	mitochondrial small ribosomal subunit
GO:0000276	7/8	0.0006	mitochondrial proton-transporting ATP synthase complex coupling factor F(o)
GO:0005747	44/44	0.0126	mitochondrial respiratory chain complex I
GO:0005742	6/6	0.024	mitochondrial outer membrane translocase complex
**Motor Neuron**			
GO:0031594	8/25	0.028	neuromuscular junction
GO:0030424	21/133	0.1525	axon
**Nucleus**			
GO:0005680	12/24	0.0167	anaphase-promoting complex
GO:0000777	18/61	0.1854	condensed chromosome kinetochore
GO:0000779	20/4	0.1989	condensed chromosome centromeric region
GO:0000783	9/9	0.1197	nuclear telomere cap complex
GO:0005643	51/67	0.2084	nuclear pore
GO:0005637	10/28	0.1318	nuclear inner membrane
**Transcriptional Complexes**			
GO:0016591	43/6	0.001	DNA-directed RNA polymerase II holoenzyme
GO:0000178	11/9	0.0025	exosome (RNase complex)
GO:0016580	10/8	0.0712	sin3 complex
GO:0005669	11/20	0.1817	transcription factor TFIID complex
**UPS**			
GO:0005832	5/7	0.0001	chaperonin-containing T-complex
GO:0000151	57/57	0.0003	ubiquitin ligase complex
GO:0000152	14/2	0.024	nuclear ubiquitin ligase complex
GO:0031461	10/8	0.0012	cullin-RING ubiquitin ligase complex
GO:0005839	20/14	0.0167	proteasome core complex
GO:0016272	8/10	0.0402	prefoldin complex
GO:0008180	8/10	0.003	signalosome

^α^Gene ontology (GO) pathway identities for cellular components and ^β^number of probes on the 4 × 44K human genome array per GO group of directly associated proteins is shown (note: one gene may be represented by different or redundant probes on the array). ^γ^FDR (q-value) at significance level q < 0.25 (25%) was determined by bootstrapping following SAFE analysis. ^δ^The table shows partial listing of representative significant ^ε^GO categories associated with cellular components (36 among 83). UPS stands for the ubiquitin/proteasome system and ER for endoplasmic reticulum.

**Table 5:** SAFE analysis of the KEGG pathway database

^**α**^**Pathway (UP)**	^**β**^**Size**	^**δ**^**q-value**	^**ε**^**GO Term**
**Disease**			
KEGG:05014	92/54	0.1831	Amyotrophic lateral sclerosis
**DNA Metabolism**			
KEGG:03030	35/36	0.2048	DNA replication
KEGG:03410	46/34	0.0003	Base excision repair
KEGG:03420	42/44	0.0758	Nucleotide excision repair
KEGG:03430	36/23	0.1367	Mismatch repair
KEGG:03440	13/28	0.0003	Homologous recombination
**RNA Metabolism**			
KEGG:03018	55/54	0.0007	RNA degradation
KEGG:03020	16/29	0.0296	RNA polymerase
KEGG:00970	18/63	0.005	Aminoacyl-tRNA biosynthesis
**Mitochondrial Function**			
KEGG:00062	5/8	0.0381	Fatty acid elongation in mitochondria
KEGG:00071	17/44	0.1726	Fatty acid metabolism
**Amino acid Metabolism**			
KEGG:00270	22/36	0.0261	Cysteine and methionine metabolism
KEGG:00280	24/44	0.025	Valine leucine and isoleucine degradation
KEGG:00290	7/11	0.0072	Valine leucine and isoleucine biosynthesis
KEGG:00310	24/44	0.0385	Lysine degradation
KEGG:00450	13/26	0.2048	Selenoamino acid metabolism
**Carbohydrate Metabolism**			
KEGG:00632	8/33	0.0003	Benzoate degradation via CoA ligation
KEGG:00640	18/32	0.0381	Propanoate metabolism
KEGG:00650	13/30	0.0003	Butanoate metabolism
KEGG:00670	8/18	0.0177	One carbon pool by folate
KEGG:00903	7/32	0.0425	Limonene and pinene degradation
KEGG:00510	31/49	0.2155	N-Glycan biosynthesis
**Cell-Cell Communication**			
KEGG:04330	35/47	0.2288	Notch signaling pathway
**Chaperone-dependent protein transport**			
KEGG:03060	13/23	0.0573	Protein export
**Neuromuscular**			
KEGG:04260	46/77	0.1493	Cardiac muscle contraction
**UPS**			
KEGG:03050	42/45	0.032	Proteasome
KEGG:04120	93/138	0.032	Ubiquitin mediated proteolysis

^α^KEGG pathway identities and ^β^number of probes of the array mapping to the pathway per number of unique genes representing the pathway is shown. FDR (q-value) at significance level p < 0.25 (25%) was determined by bootstrapping following SAFE analysis. ^ε^The table shows listing of the 27 significant KEGG pathway categories. UPS stands for the ubiquitin/proteasome system.

We found that only neurodegenerative disease pathways (4 in total) were significantly upregulated (Additional File 2). In this latter analysis, ALS was less significant than the other three neurodegenerative diseases (Huntington's disease, Parkinson's disease, Alzheimer's disease), suggesting that neurodegeneration affects lymphocytes to a greater extent than ALS-specific biological processes. Nevertheless, such interpretation has to be taken with caution. Indeed, some genes represented on the pathway maps of these other three neurodegenerative diseases are related to the UPS, cytoskeleton or dynein-dynactin complex, and therefore should be represented on the KEGG ALS pathway. However, ALS, Alzheimer's, Huntington's and Parkinson's are all neurodegenerative diseases related to aging and/or associated with mitochondrial dysfunction. For this reason, results are in alignment with the results of Saris *et al. *(2009) [11]. Prion disease, generally thought to be less related to the other neurodegenerative disorders, was found not significant (Additional File 2).

**Figure 2:**
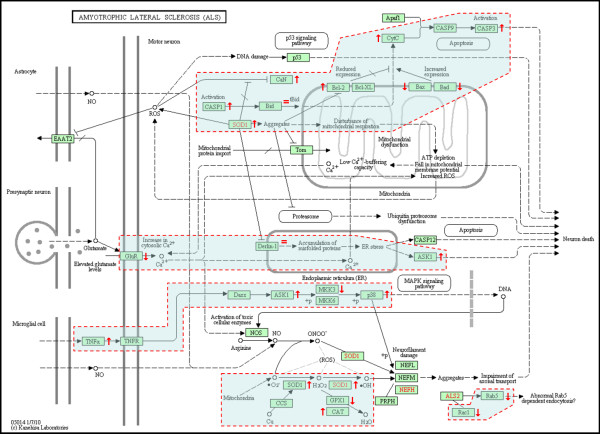
**Genes from lymphocytes of ALS patients contributing to the perturbation of the KEGG ALS pathway per SAFE analysis**. The KEGG (Kyoto Encyclopedia of Genes and Genomes) ALS pathway map relates to motor neuron degeneration in the context of a microenvironment represented by glial cells and can be found online at http://www.genome.jp/kegg/pathway/hsa/hsa05014.html. There are 54 unique gene entries (including *CASP12 *pseudogene) defined by ENTREZ identities. There are 36 protein entities represented on the map that are not all designated by official HUGO gene symbols. Red dashed areas represent subpathway modules affected by differentially expressed genes. Up or down-regulations determined following SAFE for DS7000 are shown by (↑) or (↓), unchanged is shown by (=) (fold changes in expression and HUGO gene symbols are reported in Table 6). HUGO aliases for protein entities represented on the map are as follows: **ALS2 **[*ALS2*], **Apaf1 **[*APAF1*], **ASK1 **[*MAP3K5*], **Bad **[*BAD*], **Bax **[*BAX*], **Bcl2 **[*BCL2*], **Bcl-XL **[*BCL2L1*], **Bid **[*BID*], **CaN **[*CHP*, *CHP2*, *PPP3CA*, *PPP3CB*, *PPP3CC*, *PPP3R1*, *PPP3R2*], **CASP1 **[*CASP1*], **CASP3 **[*CASP3*], **CASP9 **[*CASP9*], **CASP12 **[*CASP12*], **CAT **[*CAT*], **CCS **[*CCS*], **CytC **[*CYCS*], **Daxx **[*DAXX*], **Derlin-1 **[*DERL1*], **EAAT2 **[*SLC1A2*], **GPX1 **[*GPX1*], **GluR **[*GRIA1*, *GRIA2*, *GRIN1*, *GRIN2A*, *GRIN2B*, *GRIN2C*, *GRIN2D*], **MKK3 **[*MAP2K3*], **MKK6 **[*MAP2K6*], **p38 **[*MAPK11*, *MAPK12*, *MAPK13*, *MAPK14*], **NEFH **[*NEFH*], **NEFL **[*NEFL*], **NEFM **[*NEFM*], **NOS1 **[*NOS1*], **p53 **[*TP53*], **PRPH **[*PRPH*, *PRPH2*], **Rab5 **[*RAB5A*], **Rac1 **[*RAC1*], **SOD1 **[*SOD1*], **TNF-α **[*TNF*], **TNFR **[*TNFRSF1A*, *TNFRSF1B*], and **Tom **[*TOMM40*, *TOMM40L*].

A total of 54 unique gene IDs (including the pseudogene caspase 12) constitute the KEGG ALS pathway (hsa05014) [37] and correspond to 36 protein entities defining unique proteins or protein complexes (Figure 2). A total of 35 protein entities corresponding to 53 genes represented on the KEGG ALS pathway map (pseudogene *CASP12 *excluded) include membrane receptors, cytosolic or secreted proteins, kinases, phosphatases, proteases, and protein channels, which are likely to play a direct/indirect role in ALS pathogenesis to a variable degree at different stages that lead to motor neuron degeneration. Some protein entities may correspond to different isoforms represented by unique gene IDs. For example, calcineurin (CaN entity) may be composed by three catalytic isoforms (α,β,γ) encoded by three different chromosomes and many types of glutamate receptors may represent the GluR entity (Figure 2).

There were 23 unique genes (43%, 81 probes), the aggregate expression pattern of which contributes to the perturbation of the KEGG ALS pathway (Table 6). The number of protein entities defined by these genes and represented on the KEGG ALS pathway map was 19 out of 35 (54%) (Table 6, Figure 2). The dynamics of up- and down-regulations, assuming they are functionally effective, may be interpreted as responses to signals originating from serum or cell-cell interactions. For example upregulation of *ASK1 *(*alias MAP3K5*) may be associated with an ER-stress response that correlates with ALS progression (Figure 2). Also, assuming that transcriptional regulation produces more or less active protein with appropriate subcellular localization and in a timely manner, about half of the genes would have a negative effect on motor neuron survival while the rest would have a positive effect according to current ALS literature. This said contribution of aggregate expression to a pathway does not necessarily signify differential expression of each participant gene in terms of differences between the mean expression levels in lymphocytes from ALS *vs*. healthy controls. One limitation is that individual protein entities of the UPS, the dynein-dynactin complex, and *TARDBP*/TDP43 pathway and other elements of ALS pathogenesis are not represented on the KEGG ALS pathway, so that other potential aggregate effects relevant of ALS pathogenesis cannot be determined using the current gene set. However, pathway perturbations were determined by SAFE for genes belonging to the UPS both in terms of "biological process" and "cellular components" affected (Tables 3 and 4). Gene ontology categories corresponding to gene sets related to the UPS (AmiGO database) [38] included the following: positive or negative regulation of ubiquitin-protein ligase, proteasomal ubiquitin-dependent proteins [GO:0051443, 6 proteins; GO:0051444, 4 proteins; GO:0043161, 43 proteins] (Table 3); chaperonin-containing T-complex, ubiquitin ligase complex, nuclear ubiquitin ligase complex, cullin-RING ubiquitin ligase complex, proteasome core complex, prefoldin complex, and signalosome [GO:0005832, 7 proteins; GO:0000151, 57 proteins; GO:0000152, 2 proteins; GO:0031461, 8 proteins; GO:0005839, 14 proteins, GO:0016272, 10 proteins; GO:0008180, 10 proteins] (Table 4). Considering gene listing overlaps, UPS GO categories for biological processes and cellular components found significant by SAFE, represent altogether 234 unique proteins defined by official HUGO (Human Genome Organization) gene nomenclature. Among 220 unique genes that were differentially expressed, as determined by SAM and LIMMA, (Figure 1, Additional File 1), nine are related to the UPS, including four E3 ubiquitin ligases (*RNF149*, *TRIM22*, *UBR1*, and *UBR2*) (Table 7). Also, *ANAPC4*, *SHFM1*, *SUGT1*, *UBR1 *and *UBR2 *were represented by the UPS GO groups found significant by SAFE.

**Table 6:** Genes differentially expressed in lymphocytes from ALS patients compared to healthy controls and contributing to the KEGG ALS pathway as determined by SAFE

^**α**^**Entrez Gene ID**	^**β**^**Symbol**	^**γ**^**Description**	^**δ**^**SAFE**	^**ε**^**PS/PT**	^**λ**^**FC**	^**κ**^**Effect in ALS**			^**τ**^**LR**
57679	*ALS2*	amyotrophic lateral sclerosis 2 (juvenile)	-	n.d.	<	↓	-	[11586298]	n.d.
317	*APAF1*	apoptotic peptidase activating factor 1	-	n.d.	<	↑	-	[16046141]	n.d.
572	*BAD*	BCL2-associated agonist of cell death	+	1/1	1.38 ↓	↑	-	[19043451]	+
581	*BAX*	BCL2-associated X protein	+	10/11	1.11 ↓	↑	-	[17171827]	+
596	*BCL2*	apoptosis regulator B-cell CLL/lymphoma 2	+	10/11	1.07 ↑	↑	+	[20460269]	+
598	*BCL2L1*	inhibitor of cell death BCL2-like 1	-	n.d.	<	↑	+	[12097494]	n.d.
637	*BID*	BH3 interacting domain death agonist	+	1/2	<	↑	-	[12213439]	n.d.
834	*CASP1*	caspase 1, apoptosis-related cysteine peptidase	+	1/1	1.54 ↑	↑	-	[10764647]	-
836	*CASP3*	caspase 3, apoptosis-related cysteine peptidase	+	10/10	1.20 ↑	↑	-	[10764647]	-
842	*CASP9*	caspase 9, apoptosis-related cysteine peptidase	-	2/2	1.07 ↑	↑	-	[14657037]	-
847	*CAT*	catalase (heme containing)	+	1/1	1.46 ↑	↑	+	[8731383]	+
9973	*CCS*	copper chaperone for superoxide dismutase	-	1/1	1.08 ↓	↓	-	[17389365]	-
11261	*CHP*	calcineurin B homolog	+	1/1	1.16 ↑	↑	+	[11350981]	+
63928	*CHP2*	calcineurin B homologous protein 2	-	n.d.	<	↑	+	[12226101]	n.d.
54205	*CYCS*	cytochrome c, somatic	+	2/3	1.10 ↑	↓	-	[17454840]	+
1616	*DAXX*	death-domain associated protein	-	n.d.	<	↑	-	[12354397]	n.d.
79139	*DERL1*	degradation in endoplasmic reticulum protein 1	+	1/1	<	↑	-	[18519638]	n.d.
2876	*GPX1*	glutathione peroxidase 1	+	1/1	1.22 ↓	↓	-	[9335008]	-
2890	*GRIA1*	glutamate receptor, ionotropic, AMPA 1	-	n.d.	<	↓	-	[8981413]	n.d.
2891	*GRIA2*	glutamate receptor, ionotropic, AMPA 2	-	n.d.	<	↓	-	[8981413]	n.d.
2902	*GRIN1*	glutamate receptor, ionotropic, N-methyl D-aspartate 1	+	2/2	1.44 ↓	↓	-	[1320444]	-
2903	*GRIN2A*	glutamate receptor, ionotropic, N-methyl D-aspartate 2A	-	n.d.	<	↓	-	[8842405]	n.d.
2904	*GRIN2B*	glutamate receptor, ionotropic, N-methyl D-aspartate 2B	-	n.d.	<	↓	+	[16490316]	n.d.
2905	*GRIN2C*	glutamate receptor, ionotropic, N-methyl D-aspartate 2C	-	n.d.	<	↓	+	[11717388]	n.d.
2906	*GRIN2D*	glutamate receptor, ionotropic, N-methyl D-aspartate 2D	+	1/1	1.58 ↓	↑	+	[15152019]	-
5606	*MAP2K3*	mitogen-activated protein kinase kinase 3	+	1/1	1.34 ↓	↑	-	[17686961]	+
5608	*MAP2K6*	mitogen-activated protein kinase kinase 6	-	1/1	1.32 ↑	↑	-	[16219474]	-
4217	*MAP3K5*	mitogen-activated protein kinase kinase kinase 5	+	1/1	1.10 ↑	↑	-	[15910777]	-
5600	*MAPK11*	mitogen-activated protein kinase 11	-	n.d.	<	↑	-	[9218798]	n.d.
6300	*MAPK12*	mitogen-activated protein kinase 12	-	n.d.	<	↑	-	[9169156]	n.d.
5603	*MAPK13*	mitogen-activated protein kinase 13	-	n.d.	<	↑	-	[9218798]	n.d.
1432	*MAPK14*	mitogen-activated protein kinase 14	+	10/10	1.26 ↑	↑	-	[15910777]	-
4744	*NEFH*	neurofilament, heavy polypeptide	-	n.d.	<	↓	-	[7849698]	n.d.
4747	*NEFL*	neurofilament, light polypeptide	-	n.d.	<	↓	-	[15207859]	n.d.
4741	*NEFM*	neurofilament, medium polypeptide	-	n.d.	<	↓	-	[11732278]	n.d.
4842	*NOS1*	nitric oxide synthase 1 (neuronal)	-	n.d.	<	↑	-	[15033415]	n.d.
5530	*PPP3CA*	protein phosphatase 3, catalytic subunit, alpha isozyme	+	3/3	1.06 ↑	↓	-	[11701756]	+
5532	*PPP3CB*	protein phosphatase 3, catalytic subunit, beta isozyme	+	1/1	1.28 ↑	↓	-	[15312178]	+
5533	*PPP3CC*	protein phosphatase 3, catalytic subunit, gamma isozyme	+	1/1	<	↓	-	[15312178]	n.d.
5534	*PPP3R1*	protein phosphatase 3, regulatory subunit B, alpha	-	n.d.	<	↓	-	[11754729]	n.d.
5535	*PPP3R2*	protein phosphatase 3, regulatory subunit B, beta	-	n.d.	<	↓	-	[11754729]	n.d.
5630	*PRPH*	peripherin	-	n.d.	<	↓	-	[20363051]	n.d.
5961	*PRPH2*	peripherin 2	-	n.d.	<	↓	-	[8125718]	n.d.
5868	*RAB5A*	ras-related protein Rab-5A	+	1/1	1.11 ↓	↓	+	[11316809]	+
5879	*RAC1*	ras-related C3 botulinum toxin substrate 1	+	12/12	1.08 ↓	↓	-	[18219391]	-
6506	*SLC1A2*	sodium-dependent glutamate/aspartate transporter 2	-	n.d.	<	↑	+	[14530974]	n.d.
6647	*SOD1*	superoxide dismutase 1, soluble	+	11/11	1.24 ↑	↓	-	[20644736]	+
7124	*TNF*	tumor necrosis factor alpha	+	1/1	1.15 ↑	↑	+	[18823372]	+
7132	*TNFRSF1A*	tumor necrosis factor receptor superfamily, member 1A	-	n.d.	<	↑	-	[11917000]	n.d.
7133	*TNFRSF1B*	tumor necrosis factor receptor superfamily, member 1B	-	n.d.	<	↓	+	[11917000]	n.d.
10452	*TOMM40*	mitochondrial import receptor subunit TOM40 homolog	-	n.d.	<	↓	-	[20797528]	n.d.
84134	*TOMM40L*	mitochondrial import receptor subunit TOM40B	-	1/1	1.23 ↓	↓	-	[20797528]	-
7157	*TP53*	tumor protein p53	-	n.d.	<	↑	-	[8609941]	n.d.

Genes (53 in total, pseudogene *CASP12 *excluded) belonging to the KEGG ALS pathway (hsa05014) that describe pathogenic effects in motor neurons are defined by their official HUGO symbol. ^α^Entrez Gene accession number and ^β^HUGO symbol and ^γ^description are provided. ^δ^Genes that contribute to the KEGG ALS pathway through SAFE analysis are marked with a (+) sign and if not with a (-) sign. Number of probes significant (PS) per total number of probe signal intensity values per gene on the Agilent 4 × 44K array (PT) is shown. ^λ^FC is average fold change ALS (n = 11) compared to healthy controls (n = 11) considering one or more probe signal intensity values per gene, with upregulated genes indicated by (↑) and downregulated genes by (↓). Less than 1.05 fold changes are indicated by (<). ^κ^PMID referenced positive (+) or negative (-) effect on motor neuron survival if wild type protein activity or function is increased (↑) or normal function or activity is altered (↓). Genes that were not determined (n.d.) to contribute to the KEGG ALS pathway or with an FC < 1.05, are also referenced for known effects. ^τ^Lymphocyte response (LR) represented by the genes contributing to the KEGG ALS pathway is shown.

**Table 7:** Differentially expressed genes related to the UPS, as determined by SAM and LIMMA

^**α**^**Probe**	^**β**^**Symbol**	^**γ**^**Accession**	^**δ**^**FC**	^**ε**^**Functional Description**
A_23_P317800	*ANAPC4*	NM_013367	1.40	*anaphase promoting complex subunit 4: *Component of the anaphase promoting complex/cyclosome (APC/C), a cell cycle-regulated E3 ubiquitin ligase that controls progression through mitosis and the G1 phase of the cell cycle. The APC/C complex acts by mediating ubiquitination and subsequent degradation of target proteins: it mainly mediates the formation of 'Lys-11'-linked polyubiquitin chains and, to a lower extent, the formation of 'Lys-48'- and 'Lys-63'-linked polyubiquitin chains
A_23_P106741	*PSMD7*	NM_002811	1.41	*proteasome (prosome, macropain) 26S subunit, non-ATPase, 7: *Acts as a regulatory subunit of the 26S proteasome which is involved in the ATP-dependent degradation of ubiquitinated proteins
A_23_P120153	*RNF149*	NM_173647	1.67	*E3 ubiquitin-protein ligase RNF149: *Unknown
A_23_P42664	*SHFM1*	NM_006304	1.63	*split hand/foot malformation (ectrodactyly) type 1: *Subunit of the 26S proteasome which plays a role in ubiquitin-dependent proteolysis
A_24_P172481	*TRIM22*	NM_006074	1.55	*E3 ubiquitin-protein ligase TRIM22: *Interferon-induced antiviral protein involved in cell innate immunity. The antiviral activity could in part be mediated by TRIM22-dependent ubiquitination of viral proteins. Plays a role in restricting the replication of HIV-1, encephalomyocarditis virus (EMCV) and hepatitis B virus (HBV). Acts as a transcriptional repressor of HBV corepromoter. May have E3 ubiquitin-protein ligase activity.
A_23_P203137	*UBE4A*	NM_004788	1.34	*ubiquitin conjugation factor E4 A: *Binds to the ubiquitin moieties of preformed conjugates and catalyzes ubiquitin chain assembly in conjunction with E1, E2, and E3.
A_23_P152066	*UBR1*	NM_174916	1.33	*ubiquitin protein ligase E3 component n-recognin 1: *E3 ubiquitin-protein ligase which is a component of the N-end rule pathway. Recognizes and binds to proteins bearing specific N-terminal residues that are destabilizing according to the N-end rule, leading to their ubiquitination and subsequent degradation. Plays a critical role in chromatin inactivation and chromosome-wide transcriptional silencing during meiosis via ubiquitination of histone H2A. Binds leucine and is a negative regulator of the leucine-mTOR signaling pathway, thereby controlling cell growth.
A_23_P362637	*UBR2*	NM_015255	1.25	*ubiquitin protein ligase E3 component n-recognin 2: *Same as for *UBR1*
A_32_P178945	*YOD1*	NM_018566	1.52	*YOD1 OTU deubiquinating enzyme 1 homolog alias HIV-1-induced protease: *Hydrolase that can remove conjugated ubiquitin from proteins and participates in endoplasmic reticulum-associated degradation (ERAD) for misfolded lumenal proteins. May act by triming the ubiquitin chain on the associated substrate to facilitate their threading through the VCP/p97 pore. Ubiquitin moieties on substrates may present a steric impediment to the threading process when the substrate is transferred to the VCP pore and threaded through VCP's axial channel. Mediates deubiquitination of both 'Lys-48'- and 'Lys-63'-linked polyubiquitin chains. Able to cleave both polyubiquitin and di-ubiquitin.

A total of nine genes among 206 related to the ubiquitin/proteasome system (UPS) were significantly upregulated in lymphocytes from ALS patients compared to controls, as determined by SAM (q < 1%) and LIMMA analyses (p < 0.001). Among them, four genes encode E3 ubiquitin ligases (*RNF149*, *TRIM22*, *UBR1*, and *UBR2*) and one gene a deubiquitinase (*YOD1*). ^α^Agilent Array 4 × 44K probe IDs, ^β^gene symbol, ^γ^NCBI GenBank accession number, ^δ^fold change in expression and ^ε^GeneCard functional description (http://www.genecards.org) are provided.

### Assessment of alteration of UPS-related gene expression in lymphocytes from ALS patients based on microarray data

**Figure 3:**
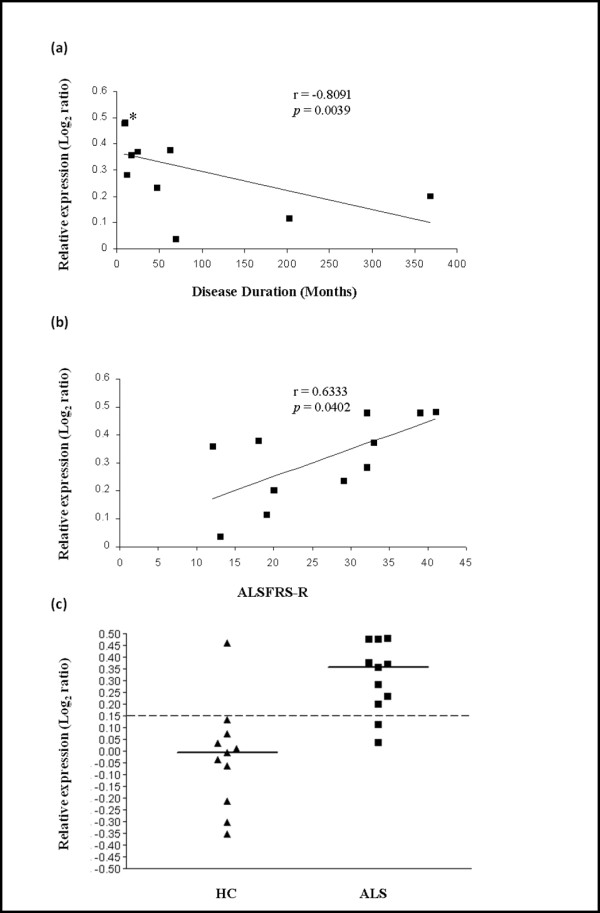
**Relationship between *UBR2 *transcriptional expression in lymphocytes from ALS patients and progression of the disease**. Expression of *UBR2 *varies inversely with the length of the disease from onset to lymphocyte gene expression testing, and varies directly with the ALS-FRS-R score. Duration of the disease from onset to sampling (*i.e. *Disease Duration) **(a) **(*three close values) or the ALSFRS-R score at the time of sampling **(b) **are indicated on the x-axis. Log_2 _ratios of expression obtained from the dual mode reference design are represented on the y axis. Dot plot **(c) **shows that with a cut-off of 0.15, discrimination between ALS patients [ALS] and healthy controls [HC] for *UBR2 *expression is achieved with *p *= 0.000953 (Fisher's exact test).

Correlation of *ANAPC4, SHFM, SUGT1, UBR1 and UBR2 *with demographic and disease parameters was determined. Among these five genes, *UBR2 *(Ubiquitin-protein ligase E3-alpha-2) encoded protein is known to act in conjunction with UBR1 in a quality control pathway for degradation of unfolded cytosolic proteins [39]. We calculated Spearman correlation between expression data and length of the disease from symptom onset and the ALS Functional Rating Scale-Revised score (ALSFRS-R) at the time of peripheral blood sampling. Significant correlation was found between *UBR2 *increased gene expression and time of disease from onset to time of lymphocyte sampling (r = -0.8091, p = 0.0039), as well as ALSFRS-R (r = 0.6333, p = 0.0402) (Figure 3, Table 8). Similar to Saris *et al. *(2009) [11], we found no correlation between the expression of these genes with gender, age at onset, age at collection, and site of onset. However, unlike Saris *et al. *(2009) [11] but similar to Zhang *et al. *(2006) [13] we present correlations of individual genes with disease duration and ALSFRS-R.

**Table 8:** Correlation between expression data of differentially expressed UPS genes and ALSFRS-R or disease duration

		Spearman Correlation	
Gene	Probe	Disease Duration	ALSFRS-R
*ANAPC4*	A_23_P317800	r = -0.0364	r = -0.2642
		p = 0.9241	p = 0.4348
*SHFM1*	A_23_P42664	r = 0.3909	r = -0.3144
		p = 0.2366	p = 0.3415
*SUGT1*	A_23_P162787	r = 0.3636	r = -0.3736
		p = 0.2731	p = 0.2608
*UBR1*	A_23_P152066	r = 0.0636	r = 0.2323
		p = 0.8603	p = 0.4854
*UBR2*	A_23_P362637	r = -0.8091	r = 0.6333
		p = 0.0039*	p = 0.0402*

*p < 0.05

### Assessment of alteration of the UPS in PBMCs from ALS patients using proteasome inhibition assays

**Figure 4:**
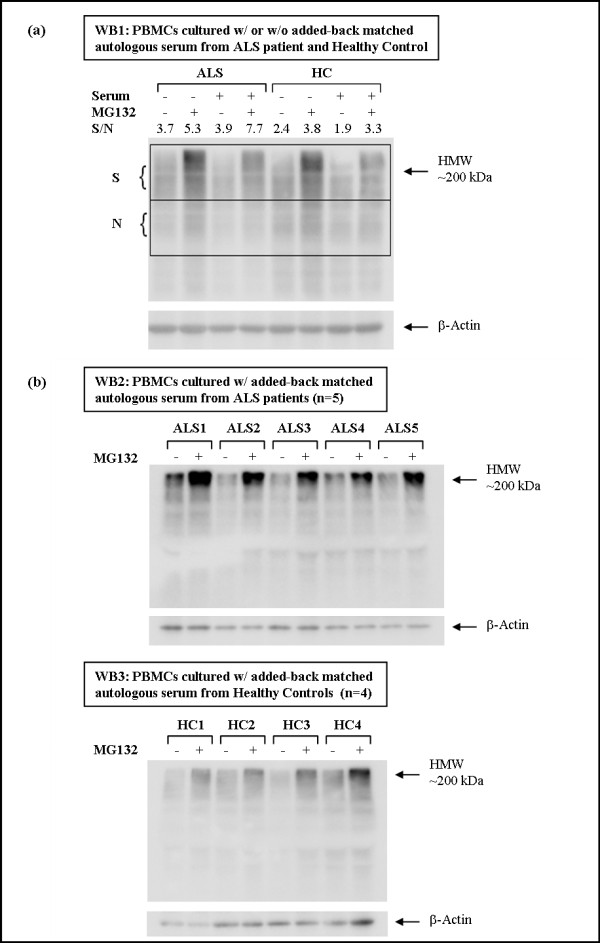
**Total ubiquitination Western blot (WB) analysis of cultured PBMCs from ALS patients and controls in the presence or absence of added-back serum for 16 hours and treated or not with proteasome inhibitor MG132 for 1.5 hr**. Comparison of PBMCs from one healthy control and one ALS patient incubated or not in the presence of added-back matched autologous serum is shown in **(a)**. Semi-quantitative Western blot analysis was performed to measure the accumulation of high molecular weight (HMW) ubiquitinated protein species in PBMCs that were prepared the same day from one healthy control and one ALS patient (WB1). A signal (S) to noise (N) ratio (S/N) was determined with ImageJ program by comparing the integrated density of two areas consistently stained throughout the membrane and visually contrasting the accumulation of HMW ubiquitinated protein species (WB1). ALS patient serum exacerbates the effects of MG132 on total ubiquitination and accumulation of HMW ubiquitinated species, while serum from healthy control mitigates these effects. Comparison of PBMCs from ALS patients (n = 5) and healthy controls (n = 4) incubated in the presence of added-back matched autologous serum is shown in **(b)**. PBMCs obtained at different times from additional ALS patients (n = 5) and healthy controls (n = 4) show similar result (WB2 and WB3).

We employed the MG132 proteasome inhibition assay to test whether the UPS transcriptional alterations described above are accompanied by ubiquitination changes at the protein level. MG132 blocks the proteolytic activity of the 26S proteasome complex reversibly, which inhibits the degradation of ubiquitin-conjugated proteins and has multiple effects including, for instance, reducing muscle atrophy associated with disuse [40] or increasing caspase-mediated generation of TDP-43 C-terminal fragments [41]. We prepared peripheral blood mononuclear cell (PBMC) short-term cultures from ALS patients (n = 6) and healthy control subjects (n = 5). High molecular weight (HMW) poly-ubiquitinated protein forms were detected in protein lysates of these PBMCs by Western blot analysis using monoclonal anti-ubiquitin antibody similarly to Jury *et al. *(2003) [42]. For PBMCs from healthy control subjects, cultured in RPMI [10% FCS] medium, accumulation of HMW poly-ubiquitinated proteins was induced by MG132 treatment, but this accumulation was partially mitigated by the supplementation of the RPMI [10% FCS] medium with matched autologous human serum at a final concentration of 20% (Figure 4). For PBMCs from ALS patients, cultured in RPMI [10% FCS] medium, accumulation of HMW poly-ubiquinated proteins was induced by MG132 treatment, and this accumulation was further increased by addition of autologous human serum from each ALS patient (Figure 4).

## Discussion

We report, for the first time, genome-wide expression profiling of purified lymphocytes from patients with amyotrophic lateral sclerosis. This study, performed with the long oligonucleotide Agilent Human Whole Genome 44 × 4K Array, demonstrates that ALS relevant differential gene expression and pathway perturbations can be identified in peripheral blood lymphocytes by a functional enrichment method such as SAFE [20] and not only in brain or spinal cord that are directly affected by the disease. In the search for blood biomarkers in neurological disorders, determination of molecular signatures or pathway alterations becomes critical in the analysis of microarray data generated from the blood compartment. This is due to the fact that at the genome-wide scale of gene expression, relevant biological differences may be modest or even negligible relative to the noise. The expression profiling studies on whole blood from ALS patients by Saris *et al. *(2009) [11] and Lin *et al. *(2009) [10] clearly illustrate the challenge for data interpretation when variations in gene expression are minimal and performed by different methods. In the first case, 2300 probe-encoded genes were differentially expressed with fold changes in expression (ALS *vs*. controls) varying from 1.015 to 1.588 (mean value ± SD = 1.097 ± 0.073). In the second case, Lin *et al. *(2009) also reported small fold changes in expression of four mitochondrial genes of the electron transport chain (*FLAD1*, *RFK*, *CYCS*, and *SDHB*). A broader range of fold changes in expression for subcultured PBMCs was reported in the work by Zhang *et al. *(2011) [13] which could be due to subculture conditions and/or the method chosen for normalization of the microarray data [43,44]. In our study, fold changes in expression for the genes found significant by SAM and LIMMA (*i.e. *DS3500), varied from 1.244 to 3.422 (mean value ± SD = 1.556 ± 0.28). Therefore, one may not expect to correlate differential expression by qRT-PCR for many genes due to the large sample size required to eventually confirm small changes in expression. This problem is partially circumvented by global pathway analysis methods. Many differentially expressed genes identified by SAM and LIMMA may be subjectively placed in the context of ALS pathogenesis. In addition, there was little overlap with 166 genes that were found associated with ALS to a variable degree in several single-gene and genome-wide association studies (GWAS). For instance, *TARDBP*, *SOD1*, *KIFAP3 *and *COX7C *were differentially expressed in our study. *TARDBP *and *SOD1*, clearly associated with ALS pathogenesis, have also been identified by various genetic analyses for their association with ALS. The fact that SAFE, LIMMA, and SAM identified *SOD1 *mRNA upregulation to be significant confirms findings by Gagliardi *et al. *(2010). Gagliardi *et al. *showed that *SOD1 *mRNA levels were increased in spinal cord, brain stem, and lymphocytes of sporadic ALS patients, but did not correlate with gender, age or duration of the disease [12].

For the first time, gene expression data from the blood compartment from sporadic ALS patients could be associated with the KEGG ALS disease pathway and KEGG disease pathways of neurodegenerative disorders such as Alzheimer's, Parkinson's and Huntington's diseases. Considering that global genome-wide subtle changes in gene expression were used for this determination, this result is rather unexpected. Protein activity changes that are caused by the presence of the disease are generally not expected to consistently correspond to transcriptional regulations. Our use of purified lymphocytes has likely provided a better dataset to study ALS-specific signature in the blood compartment as opposed to total blood.

However, because of the small sample size of our study (n = 22) and because ALS is a heterogeneous disease, it is not possible to capture the breadth of the disease process occurring during onset and progression of the disease. In addition, disease responses in lymphocytes may not mirror many of the disease processes occurring in brain, which depend on the alteration of the blood brain barrier and the microenvironment represented by glia and microglia. Furthermore, assuming that transcriptional regulation produces more or less active protein with appropriate subcellular localization and in a timely manner, about half of the genes based on their expression would have a negative effect on motor neuron survival while the rest would have a positive effect according to current ALS literature (Table 6). This clearly indicates very limited replication of processes occurring in brain or spinal cord of ALS patients. Thus, while similar pathways are affected in motor neurons and lymphocytes due to a possible systemic common cause(s), it is expected that some responses may differ in their details possibly reflecting differential susceptibility.

In our pathway analysis of the dataset DS7000 generated with Agilent Human Whole Genome 4 × 44K Array, SAFE identified alteration of gene expression pertaining to gene ontology (GO) categories relevant to ALS pathogenesis (and/or other neurological diseases), such as DNA metabolism, RNA splicing, mitochondrial function, oxidation, ER and Golgi functions, UPS, neurological function, post-translational modification and viral infection. These results are consistent with findings by Saris *et al. *(2009) that were determined by whole blood RNA profiling [11]. However, following pathway analysis using SAFE, we went further in the analysis of transcriptional alterations of the UPS by identifying a correlation between the expression of differentially expressed individual UPS-related genes and the time of presence of the disease or the ALSFRS-R. Indeed, whole exome sequencing identified mutations in the gene encoding valosin-containing protein (VCP), a key component of the UPS, as a cause of familial ALS, demonstrating that disturbances of UPS function may be closely linked to ALS pathogenesis [45]. A total of nine differentially expressed genes, were related to the UPS including four ubiquitin ligases representative of UPS GO groups identified by SAFE (*ANAPC4*, *SHFM1*, *UBR1*, and *UBR2*). Differential expression of the "N-end rule" ubiquitin ligase *UBR2 *gene [46] in lymphocytes from ALS patients was found to correlate with disease duration and ALSFRS-R at the time of sampling. Although, overall *UBR2 *mRNA expression is upregulated in ALS patients compared to healthy controls, a decrease in expression correlated with more advanced stage or severity. This apparent paradox can be explained by the possibility that an initial disease process to which healthy controls are never exposed, causes an initial upregulation of *UBR2 *mRNA expression which then declines as the disease progresses with increasing impairment of the UPS machinery. One possible mechanism of action of E3 ubiquitin ligases UBR1 and UBR2 could be to facilitate targeting of foldable conformers to the proteasome [39] and to provide protection against toxicity of (unknown) misfolded proteins that accumulate during the disease course in lymphocytes from ALS patients. This mechanism is similar to the E3 ubiquitin ligase dorfin (encoded by *RNF19A*) that prevents mutant SOD1-mediated neurotoxicity and improves symptoms in the transgenic G93A SOD1 mouse model [47,48]. Indeed, the presence of some cellular toxicity in PBMCs was shown by De Marco *et al. *(2010) [49] who determined that the cytoplasmic fraction of TDP-43 in circulating PBMCs of sporadic and familial ALS patients was increased. In addition, by analogy with mutant SOD1-mediated toxicity, human wild-type TDP-43-mediated neurotoxicity might be partially alleviated by co-expression with ubiquilin 1 (encoded by *UBQLN1*) involved in autophagy and proteasome targeting [50,51]. Moreover, mutations in ubiquilin 2 (encoded by *UBQLN2*) have been associated with X-linked juvenile ALS and adult sporadic ALS [52]. Ubiquilins bind to both ubiquitin ligases and the proteasome, providing a connector function within the UPS [53].

Our proteasome inhibition assays also indicate that lymphocytes from ALS patients exposed to serum factors and metabolites *in vivo *have acquired new properties with regard to the UPS and other pathways that are normally perturbed in degenerating motor neurons. In this respect, the study by Watanabe *et al. *(2010) [54], showing that metabolic alterations of the UPS may take place in the skin of ALS patients, follows the same paradigm. In addition, using short-term PBMC cultures Zhang *et al. *(2011) [13] showed that monocytes in ALS patients have acquired unique properties that relate to neuroinflammation and innate immunity.

## Conclusions

Our approach demonstrates that subtle changes in gene expression measured by Agilent Human Whole Genome 4 × 44K Array may be interpreted objectively. Without underestimating the complexity of ALS pathogenesis, our analyses with these arrays identify multiple new directions worth further investigation, including systemic UPS pathway alterations, in the search of biomarkers associated with the cause(s) or the progression of ALS. Overall, it remains to be determined which properties the circulating lymphocytes acquire by long distance signaling in the peripheral blood system, and which properties they acquire by local signaling or local cell-cell contact due to trafficking of the lymphocytes at the sites of neurodegeneration in brain or spinal cord.

## Competing interests

The authors declare that they have no competing interests.

## Authors' contributions

J-LM conceived the study and designed the microarray experiments and proteasome inhibition experiments, conducted MIDAS-TM4 data normalization, SAM and LIMMA analyses, and Western blot analyses with ImageJ program, and wrote the manuscript. J-LM isolated lymphocytes from ALS patients and controls which were lysed in TriZol prior to shipping to Cogenics, Inc. for microarray experiments with Agilent Human Whole Genome 4 × 44k Arrays. FAW and ZL performed the SAFE analyses and compared MIDAS-TM4 normalization to several alternative methods of normalization (MIDAS-TM4 producing best normalization), verified SAM and LIMMA analyses. AEP isolated PBMCs from ALS patients and healthy controls, carried out the proteasome inhibition assays and Western blotting. BRB coordinated ALS patients and healthy controls sample collection, verified diagnosis and disease characteristics at the time of lymphocyte sampling and contributed to revisions of the manuscript and to the emphasis on clinical significance of the results. All authors have read and approved the final manuscript.

## Pre-publication history

The pre-publication history for this paper can be accessed here:

http://www.biomedcentral.com/1755-8794/4/74/prepub

## Supplementary Material

Additional file 1 **SAM analysis (q < 1%) and LIMMA analysis conducted independently using DS7000**. Probe set IDs, gene symbol, GenBank NCBI accession number, fold change (FC) ALS *vs*. healthy controls, local FDR (q value in %), LIMMA significance p value and q value, and log_2 _ratios of normalized expression data and gene descriptions are shown. FC >1 signifies higher expression in the ALS group. Click here for file

Additional file 2 **SAFE results of the KEGG disease pathways**. Raw SAFE data are presented for the 25 disease pathways (gene set size 5-100) and 34 disease pathways (including gene sets with >100 genes) analyzed using the DS7000 microarray dataset. These pathways represent cancer, circulatory, genetic, immune, neurological and urological diseases. Click here for file
